# The sweet tabaiba or there and back again: phylogeographical history of the Macaronesian *Euphorbia balsamifera*

**DOI:** 10.1093/aob/mcae001

**Published:** 2024-01-10

**Authors:** Mario Rincón-Barrado, Tamara Villaverde, Manolo F Perez, Isabel Sanmartín, Ricarda Riina

**Affiliations:** Real Jardín Botánico (RJB), CSIC, Madrid, 28014, Spain; Centro Nacional de Biotecnología (CNB), CSIC, Madrid, 28049, Spain; Universidad Rey Juan Carlos (URJC), Área de Biodiversidad y Conservación, Móstoles, 28933, Spain; Institut de Systématique, Evolution, Biodiversité (ISYEB – URM 7205 CNRS), Muséum National d’Histoire Naturelle, SU, EPHE & UA, Paris, France; Real Jardín Botánico (RJB), CSIC, Madrid, 28014, Spain; Real Jardín Botánico (RJB), CSIC, Madrid, 28014, Spain

**Keywords:** Africa, back-colonization, Canary Islands, convoluted neural networks, Hyb-Seq, island colonization models, population genomics, Rand Flora, single nucleotide polymorphism

## Abstract

**Background and Aims:**

Biogeographical relationships between the Canary Islands and north-west Africa are often explained by oceanic dispersal and geographical proximity. Sister-group relationships between Canarian and eastern African/Arabian taxa, the ‘Rand Flora’ pattern, are rare among plants and have been attributed to the extinction of north-western African populations. *Euphorbia balsamifera* is the only representative species of this pattern that is distributed in the Canary Islands and north-west Africa; it is also one of few species present in all seven islands. Previous studies placed African populations of *E. balsamifera* as sister to the Canarian populations, but this relationship was based on herbarium samples with highly degraded DNA. Here, we test the extinction hypothesis by sampling new continental populations; we also expand the Canarian sampling to examine the dynamics of island colonization and diversification.

**Methods:**

Using target enrichment with genome skimming, we reconstructed phylogenetic relationships within *E. balsamifera* and between this species and its disjunct relatives. A single nucleotide polymorphism dataset obtained from the target sequences was used to infer population genetic diversity patterns. We used convolutional neural networks to discriminate among alternative Canary Islands colonization scenarios.

**Key Results:**

The results confirmed the Rand Flora sister-group relationship between western *E. balsamifera* and *Euphorbia adenensis* in the Eritreo-Arabian region and recovered an eastern–western geographical structure among *E. balsamifera* Canarian populations. Convolutional neural networks supported a scenario of east-to-west island colonization, followed by population extinctions in Lanzarote and Fuerteventura and recolonization from Tenerife and Gran Canaria; a signal of admixture between the eastern island and north-west African populations was recovered.

**Conclusions:**

Our findings support the Surfing Syngameon Hypothesis for the colonization of the Canary Islands by *E. balsamifera*, but also a recent back-colonization to the continent. Populations of *E. balsamifera* from northwest Africa are not the remnants of an ancestral continental stock, but originated from migration events from Lanzarote and Fuerteventura. This is further evidence that oceanic archipelagos are not a sink for biodiversity, but may be a source of new genetic variability.

## INTRODUCTION

**Fig. 1.**
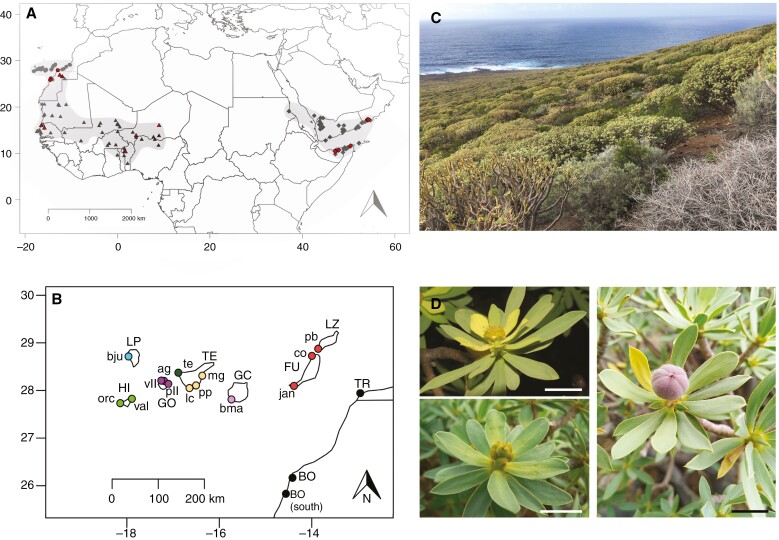
Information about the focal taxa, sampling and study area. (A) Map showing the distribution of *Euphorbia balsamifera* (circles), *Euphorbia adenensis* (diamonds) and *Euphorbia sepium* (triangles) (modified from Riina *et al.*, 2021); red points indicate samples from continental Africa and the southern Arabian Peninsula included in our analyses. (B) Map of Canary Islands and the adjacent African coast, showing the populations of *E. balsamifera* sampled in our study; colours represent different populations. (C) Tabaibal cardonal dominated by succulent shrubs of *E. balsamifera* in Teno, western Tenerife. (D) Male and female inflorescences (cyathia) and fruit of *E. balsamifera*; clockwise: male cyathium, fruit and female cyathium (photographs by the authors). Scale bars: 1 cm. Abbreviations: BO, Boujdour (Western Sahara); FU co, Fuerteventura, Malpaís de Cotillo; FU jan, Fuerteventura, Jandía; GC bma, Gran Canaria, El Medio Almud; GO ag, La Gomera, Agulo, La Gomera; GO pll, La Gomera, Punta Llana; GO vll, La Gomera, Valle Hermoso; HI val, El Hierro, Valverde; LP bju, La Palma, Barranco del Jurado; LZ pb, Lanzarote, Playa Blanca; TE lc, Tenerife, Los Cristianos; TE mg, Tenerife, Malpaís de Güímar; TE pp, Tenerife, Punta Prieta; TE te, Tenerife, Teno Bajo; TR, Tarfaya (Morocco).

The tabaibal cardonal is one of the most iconic and widespread plant formations in the Canary Islands. It consists of a shrubland dominated by succulent dendroid shrubs and cactiform species of the genus *Euphorbia* found in the coastal parts of the islands (del Arco Aguilar and Rodríguez Delgado, 2018). Together with the cactiform ‘cardón canario’ (*Euphorbia canariensis* L.), the succulent shrub *Euphorbia balsamifera* Aiton is one of the most important plant species in this ecosystem (Goday and Chueca, 1964; Henríquez *et al.*, 1986; Berg, 1990). This dioecious species, commonly known as ‘tabaiba dulce’ (sweet tabaiba), is found on all islands and islets of the Canarian Archipelago and in continental enclaves scattered along the Moroccan and Western Saharan coasts (Fig. 1; Riina *et al.*, 2021). As in most Euphorbiaceae, the fruits of *E. balsamifera* are explosive capsules that are effective only for local dispersal; seeds are secondarily dispersed by granivorous birds (Berg, 1990). The sister species of *E. balsamifera* is *Euphorbia adenensis* Deflers (as *E. balsamifera* subsp. *adenensis*; Peirson *et al.*, 2013; Villaverde *et al.*, 2018), distributed on the eastern side of the Sahara Desert, along the coasts of the Red Sea, the Horn of Africa, southern Arabia and Socotra Island (Riina *et al.*, 2021). *Euphorbia sepium* N.E.Br., in turn, is sister to these two species (Villaverde *et al.*, 2018), distributed in the western Sahel, from Niger to Western Sahara (Riina *et al.*, 2021). *Euphorbia balsamifera* and its two sister taxa, *E. adenensis* and *E. sepium* are diploid species (Molero *et al.*, 2002).

Because of its wide and disjunct geographical distribution on both sides of northern Africa, the clade formed by *E. balsamifera*, *E. adenensis* and *E. sepium* has been considered as part of the Rand Flora (Pokorny *et al.*, 2015). This term describes a biogeographical pattern in which sister species, clades or genera that belong to unrelated angiosperm families appear distributed in the continental margins of Africa and adjacent islands: the Macaronesian archipelagos, the Horn of Africa and eastern Africa, the southern Arabian Peninsula, the Socotra Archipelago and southern Africa. The origin of the Rand Flora has been attributed to climatic extinction, associated with the aridification trend that extended from the Early Miocene to the Plio-Pleistocene, leaving the surviving lineages isolated on opposite sides of the African continent (Christ, 1892; Andrus *et al.*, 2004; Sanmartín *et al.*, 2010; Pokorny *et al.*, 2015).

In many Rand Flora lineages, the north-western element is represented only by the Macaronesian endemic (Mairal *et al.*, 2015a; Culshaw *et al.*, 2021; Rincón-Barrado *et al.*, 2021). The few exceptions include the genus *Kleinia*, with two sister species occurring in north-west Africa and the Canary Islands, respectively (Pokorny *et al.*, 2015; Rincón-Barrado *et al.*, 2021), and *E. balsamifera*, which represents the only example of this distribution at the species level (Fig. 1). According to the ‘climatic extinction hypothesis’, the north-western African species/populations of *Kleinia* and *E. balsamifera* would represent the remnants of the ancestral Rand Flora distribution, which once extended across northern Africa and became fragmented by the appearance of climatic barriers (i.e. the Sahara Desert) and the ensuing geographical extinction, leaving relictural lineages at ‘refugia’ on the west and east coasts of Africa (Mairal *et al.*, 2015a; Pokorny *et al.*, 2015). The Canarian endemic would have originated from an overwater dispersal from the north-west African ancestral refugium (Mairal *et al.*, 2015a). In the first phylogenomic study of *E. balsamifera* and allies, Villaverde *et al.* (2018) found evidence for this hypothesis, showing the continental (north-west African) samples of *E. balsamifera* forming either a sister clade or a grade relative to the Canarian populations (although this relationship was not supported in all analyses; see below).

The sampling by Villaverde *et al.* (2018) consisted of a combination of silica-preserved material and dried herbarium samples: for example, *E. sepium* and *E. adenensis* were represented by old herbarium material (except for one silica sample of *E. adenensis*), whereas samples of *E. balsamifera* were all freshly collected, with the exception of three samples from Morocco and Western Sahara, which came from old herbarium material. Sequencing herbarium material is a great challenge, because the extracted DNA is often fragmented, found in very low quantities and/or contaminated with base substitutions (Drábková *et al.*, 2002; Staats *et al.*, 2011; Sayyari *et al.*, 2017; Bakker, 2018; Forrest *et al.*, 2019). However, Villaverde *et al.* (2018) used a genomic technique termed hybrid sequencing with genome skimming (Hyb-Seq) (Weitemier *et al.*, 2014), which has been argued to be efficient with degraded, fragmented DNA extractions (e.g. Villaverde *et al.*, 2018, 2020; Siniscalchi *et al.*, 2019; Gardner *et al.*, 2021).

In the study by Villaverde *et al.* (2018), most of the Hyb-Seq sequences obtained from herbarium material were associated with long terminal branches, which Sayyari *et al.* (2017) linked to fragmentary DNA and the inference of erroneous or uncertain phylogenetic relationships. This was the case for the three north-western African samples of *E. balsamifera*, which were subtended by long branches and embedded within a Canarian clade in the maximum likelihood (ML) concatenated analyses but placed as sister to all the Canarian populations in the multispecies coalescent analysis. Villaverde *et al.* (2018) attributed this difference to long-branch attraction, which affects supertree approaches more severely (Liu *et al.*, 2014; Sayyari *et al.*, 2017). They concluded that the position of the continental populations as early-diverging lineages within *E. balsamifera* was the correct one and that this species thus represents bona-fide evidence of the climatic extinction hypothesis.

The colonization and diversification history of volcanic oceanic archipelagos, such as the Canary Islands, has often been explained by the stepping-stone colonization (SSC) model. This model, based on studies of the Hawaiian Archipelago (Wagner *et al.*, 1995), proposes that the pattern of island colonization follows the sequence of island emergence, with geologically younger islands being more recently colonized than the older ones (Sanmartín *et al.*, 2008). In the case of the Canary Islands, colonization would start in the oldest islands, Lanzarote and Fuerteventura, closest to north-west Africa, and proceed to the central islands, Gran Canaria, Tenerife and La Gomera, ending in the westernmost islands, La Palma and El Hierro, which are the youngest and furthest from the African continent (van den Bogaard, 2013). However, patterns of colonization in the Canary Archipelago rarely fit the SSC model (Sanmartín *et al.*, 2008; Shaw and Gillespie, 2016). In fact, it has been dated that the origin of most groups of the Canary flora is much later than the formation of the eastern and central islands (Fernández-Palacios *et al.*, 2011; Martín-Hernanz *et al.*, 2023); therefore, the main factor involved in the colonization of the Canary Islands would be the distance from the mainland, and not the age of the islands. Many animal and plant lineages show a phylogeographical pattern compatible with an initial colonization of the island of Tenerife, the largest and highest in the Archipelago, followed by eastward dispersal towards the islands of Gran Canaria, Lanzarote and Fuerteventura and by westward dispersal, colonizing the islands of La Gomera, La Palma and El Hierro. This has resulted in the observed east–west phylogeographical structure, with Tenerife (and sometimes Gran Canaria) occupying a central position, as centres of dispersal (Sanmartín *et al.*, 2008).

This colonization pattern observed at the species level, which we term here the central island hub (CIH) hypothesis, stands in contrast to the finding of higher values of population genetic diversity in Fuerteventura and Lanzarote (Caujapé-Castells *et al.*, 2011), even in clades with a phylogeographical history compatible with the CIH model (Kim *et al.*, 1998; González-Pérez *et al.*, 2004; Oliva-Tejera *et al.*, 2005). To explain this incongruence between species- and population-level patterns of genetic variance, Caujapé-Castells *et al.* (2011, 2017) proposed the surfing syngameon hypothesis (SSH). This model posits that the initial colonization of the archipelago followed the SSC model, with an east-to-west progression. Later, aridification waves during the Pleistocene, which affected more severely north-west Africa (Aït Hssaine and Bridgland, 2009) and the eastern Canary Islands (Garcia-Verdugo *et al.*, 2019), could have led to the extinction of species and geographical extirpation of populations in these areas, favouring the recolonization by propagules from the central island(s) of Tenerife (and Gran Canaria). The end result was an increase in the genetic diversity of the eastern Canary Islands and the replacement of the initial, east-to-west nested colonization pattern by a central-east/west phylogeographical structure (Caujapé *et al.*, 2011).

The pattern found by Villaverde *et al.* (2018) in *E. balsamifera* resembles the CIH model, with an east/west split among the islands, which also divides Tenerife into two halves. Conversely, the phylogenetic position of the north-west African populations, as either early diverging or embedded within the Canarian clade, fits the SSH model better. Back-colonization to north-west Africa (Caujapé-Castells *et al.*, 2011) has been inferred in other Canarian endemics (e.g. Mort *et al.*, 2002; Carine *et al.*, 2004; Kim *et al.*, 2008; Hutsemekers *et al.*, 2011), supporting the idea that the Canary Islands did not behave as geographical ‘cul-de-sacs’, but instead might have contributed to the biodiversity of the African flora (Patiño *et al.*, 2015). Although the study by Villaverde *et al.* (2018) included numerous silica-preserved samples of Canarian populations of *E. balsamifera*, these represented only three of the seven major islands in the archipelago (Tenerife, Gran Canaria and La Gomera). Also, Villaverde *et al.* (2018) used DNA sequences only for phylogenetic inference, although an advantage of Hyb-Seq is the possibility to extract single nucleotide polymorphisms (SNPs) directly from the target loci (Andermann *et al.*, 2018). The use of phased SNP data allows researchers to adopt population-genomic approaches for estimating patterns of admixture and genetic diversity statistics, and model-based phylogeographical approaches for inferring the history of colonization events (Helmstetter *et al.*, 2020). Diffusion approaches adapted from virus phylodynamics (Lemey *et al.*, 2009; Culshaw *et al.*, 2021) and likelihood-free approximate Bayesian computation (ABC) methods (Beaumont, 2010; Mairal *et al.*, 2018) are two model-based statistical approaches that have been used to infer the phylogeographical history of Rand Flora taxa. In recent years, deep learning techniques based on convolutional neural networks (CNNs) have been introduced as a new approach to uncover patterns of genetic variation from gene genealogies and to discriminate statistically between alternative phylogeographical scenarios (Chan *et al.*, 2018; Flagel *et al.*, 2019). Convolutional neural networks present the advantage over ABC methods of capturing all the relevant information contained in the data without the need to use summary statistics (Flagel *et al.*, 2019).

Here, we make use of these advances and of a denser, expanded geographical sampling (relative to Villaverde *et al.*, 2018) of *E. balsamifera* and its sister species, including new silica-preserved individuals of *E. sepium* and *E. balsamifera*, in order to: (1) test the persistence of relict populations of *E. balsamifera* in the continent (north-west Africa) vs. climatic extinction in north-west Africa and a posterior back-colonization to the continent hypothesis, by building a more robust population-level phylogeny of *E. balsamifera* and allies; and (2) discriminate among alternative colonization and diversification scenarios of *E. balsamifera* in the Canary Islands (the CIH and SSH models and the possibility of back-colonization), using CNNs and a large SNP dataset extracted from the target loci. For the latter, we expanded the sampling within and among islands, including material from the previously unsampled islands of Fuerteventura, Lanzarote and El Hierro. Finally, (3) we assessed the impact of degraded DNA in the Hyb-Seq technique and how this can affect the reconstruction of relationships at both micro- and macro-evolutionary levels.

## MATERIALS AND METHODS

### Taxon and population sampling

Thirty-two samples of *E. balsamifera* collected in silica gel from seven localities in the islands of Fuerteventura, Lanzarote, Tenerife, La Gomera, La Palma and El Hierro in 2018 and 2019, and 15 samples collected from four localities along the coasts of Morocco (Tarfaya) and Western Sahara (north and south of Boujdour) in 2018 were sequenced for this study. In addition, we included four newly collected and silica-dried samples of *E. sepium* from Western Sahara.

The dataset newly generated in this study was complemented by 106 samples from Villaverde *et al.* (2018): 74 samples of *E. balsamifera* [one continental sample (IS401) was excluded based on the high percentage of missing data], 18 of *E. adenensis*, eight of *E. sepium* and two of other species in *Euphorbia* section *Balsamis* (*Euphorbia larica* Boiss. and *Euphorbia masirahensis* Ghaz.), which were used as the outgroup. In total, our taxon sampling included 120 individuals of *E. balsamifera* from all the Canary islands and from coastal north-west Africa, 18 of *E. adenensis* and 12 of *E. sepium*. Of these, 31 were herbarium material and 132 were silica-dried samples. Information about the provenance of samples and additional collection data are included in the Supplementary Data (Table S1).

### Probes and sequencing

Genomic DNA extractions were performed on silica-dried samples using the CTAB method (Doyle and Doyle, 1987). The DNA concentrations obtained are given in the Supplementary Data (Table S1). Samples were sonicated using Covaris E220 to obtain the targeted 550-bp-long fragments. The TruSeq Nano HT DNA Kit (Illumina Inc., San Diego, CA, USA) was used for library preparation, with one-third of the volumes recommended by the manufacturer. The indexed samples were pooled with equal quantities (between 12 and 16 samples per pool, equimolar per 1000 ng), following taxonomic criteria (by species) and geographical criteria (from populations of the same islands or nearby geographical areas).

For the enrichment of low-copy orthologous nuclear genes, we used the Euphorbiaceae kit developed by Villaverde *et al.* (2018), produced by Arbor Bioscience, following the manufacturer’s protocol. This kit was based on the transcriptome of *Euphorbia mesembryanthemifolia* Jacq. and *Euphorbia pekinensis* Rupr. and the proteome of *Ricinus communis* L., for a total target of 431 genes. Sequencing was performed in an Illumina MiSeq 600 at the genomics unit of the Centro Nacional de Investigaciones Cardiovasculares Carlos III (CNIC). The raw reads are available in GenBank under BioProject PRJNA1076475.

### Data processing

We used TRIMMOMATIC (Bolger *et al.*, 2014) to filter sequences according to their quality and to remove adapter sequences. The HybPiper 1 pipeline (Johnson *et al.*, 2016) was used to assemble read fragments, using default settings and ‘-bwa’. HybPiper 1 uses SPAdes v.3.13.1 (Bankevich *et al.*, 2012) to assemble the sequences and eliminates introns with exonerate v.2.2 (Slater and Birney, 2005). The percentage of capture can be found in the Supplementary Data (Fig. S1; Tables S2 and S3). Following this process, we obtained 431 exon matrices. Genes suspected to have paralogue sequences were investigated further using the paralogue investigator tool in HybPiper. We excluded all the matrices in which three or more possible paralogue sequences were present. This resulted in 401 exon matrices. The gene exon matrices were aligned using MAFFT v.7.310 (Katoh and Standley, 2013). Matrices with poor alignment quality (<15 % of identical sites and pairwise alignment <65 %) were removed from further analyses, leaving a final dataset of 298 gene matrices with exon information and a concatenated total length of 511 697 bp. The percentage of missing data, of each nucleotide type and of GC content per sample, is given in the Supplementary Data (Table S4).

These 298 matrices were analysed using two approaches. The first approach used RaxML v.8.2.11 (Stamatakis, 2014) with the GTRCAT model, 200 bootstraps and a slow ML optimization (‘-fa’ option) to obtain a gene tree for each exon matrix. These trees were analysed with ASTRAL v.2.5.7.3 (Mirarab and Warnow, 2015) to estimate a species-level phylogenetic tree under the multispecies coalescent model, using the default settings. Clade support was estimated by calculating local posterior probabilities (LPPs). For the second approach, the gene matrices were concatenated using Geneious (Kearse *et al.*, 2012), and a phylogenetic tree was constructed using IQ-TREE v.1.6.1 (Nguyen *et al.*, 2015) with the following options: 1000 ultrafast bootstrap (Hoang *et al.*, 2018; ‘-bb’ option), standard model selection (Kalyaanamoorthy *et al.*, 2017; ‘-m TEST’ option) and SH-like approximate likelihood ratio test (SH-aLRT; ‘-alrt 1000’ option).

The same process was applied to the supercontigs matrices (including both introns and exons) retrieved with HybPiper. Matrices with <20 % of identical sites were discarded, resulting in 217 supercontig matrices with a concatenated total length of 816 562 bp. As with the exon data, information regarding the percentage of missing data, of each nucleotide type and of GC content per sample can be found in the Supplementary Data (Table S5). All the analyses were performed using the same procedures as with the exon matrices. Additionally, owing to the unusual length of the terminal branch of sample IS459 (and, to a lesser extent, IS460) and the lowest percentage of captured sequences in comparison to the rest of the samples of the same species, we decided to repeat all the analyses without these two samples from the study by Villaverde *et al.* (2018). The results (Supplemenatary Data Figs S2–S5) showed that removal of these samples did not alter the phylogenetic relationships among the remaining samples, with identical topology for the rest of the tree. Thus, we maintained these samples in our dataset for all subsequent analyses.

Hyb-Seq allows the off-target capture of plastid genes. However, we did not analyse plastid data in this study because of the low percentage of capture of plastid genes obtained by Villaverde *et al.* (2018), which prevented us from joining two plastid datasets of uneven quality. Although the phylogenetic relationship between *E. sepium*, *E. balsamifera* and *E. adenensis* recovered from nuclear genes was also obtained with the incomplete/low-quality plastid data in the study by Villaverde *et al.* (2018), the latter proved insufficient to resolve intraspecific relationships within *E. balsamifera* populations sampled in that study.

### Divergence time estimation

Lineage divergence times were estimated in BEAST v.1.8 (Drummond *et al.*, 2012) using the supercontig matrices. A reduced dataset with only nine individuals was used for a first analysis (species-level dating): three from *E. balsamifera*, two from *E. sepium*, two from *E. adenensis*, one from *E. larica* and one from *Euphorbia hadramautica*. Matrices with poor alignment (<15 % of identical sites and pairwise alignment <65 %) were eliminated, leaving a dataset with 296 loci. The number of loci matrices used in the dating analysis was higher than in the phylogenetic analyses described above (296 vs. 217) because in the former case we included samples with a higher percentage of identical sites. The GTR substitution model was implemented, and a birth–death process with incomplete taxon sampling (Stadler, 2009) was used as tree-growth prior. We did not force the monophyly of any clade. We estimated divergence times under a random clock model, which allows the rate of molecular clock to vary at discrete time points using a truncated piecewise compound Poisson process (Drummond and Suchard, 2010). The root node was calibrated, using a secondary calibration point from the study by Horn *et al.* (2014), modelled as a normal distribution with mean 18.21 Ma and s.d. (4 Ma), which corresponds to the divergence of *E. hadramautica* from the remaining species in our dataset. We ran a Bayesian Markov chain Monte Carlo (MCMC) analysis for 200 million generations, sampling every 1000th generation. Tracer v.1.7.2 was used to verify the MCMC stationarity and that the effective population size for all parameters reached values >200 (Rambaut *et al.*, 2018). The resulting trees and parameters were summarized with TreeAnnotator v.1.8.0 (Drummond, 2007). FigTree v.1.4.2 (Rambaut *et al.*, 2014) was used to visualize and edit the maximum clade credibility tree.

Population divergence within *E. balsamifera* was estimated with a reduced sample dataset, using 25 individuals from the seven Canary Islands and four individuals from populations from north-west Africa. A matrix of supercontigs with 365 loci was used (the increase in the number of loci was attributable to the use of individuals with the highest percentage of capture). We used the constant coalescent model as the tree prior, and a normal distribution prior of 2.25 ± 0.85 Ma was used for calibrating the root node; this age estimate was obtained from the species-level dating analysis described above. The MCMC searched for 10 million generations, and the results were evaluated and summarized using the same steps and software indicated above.

### Single nucleotide polymorphism calling procedure

The SECAPR pipeline (Andermann *et al.*, 2018) was used to generate an SNP dataset for *E. balsamifera*. In the first step, SECAPR assembles the DNA reads into *de novo* contigs, instead of using reference sequences (as in HybPiper); it then filters the target sequences using the reference sequences used to design the hybridization kit. Next, the pipeline creates an aligned matrix with the sequences of all individuals and generates a consensus sequence per locus. This consensus sequence is used as a pseudo-reference to reassemble the reads, increasing the coverage of the sequences. With the sequences of each individual assembled, SECAPR phases the reads into two different sequences, one for each allele, then extracts the SNPs from these phased sequences. We modified the original pipeline, skipping the first step, the *de novo* DNA read assembly, and instead used the 401 exon sequences obtained from HybPiper previously. HybPiper sequences were then aligned in SECAPR to obtain a consensus sequence per locus. In this way, we ensured that the DNA aligned sequences used for SNP calling did not contain assembling errors or paralogues, while skipping a computationally intensive step in SECAPR. Reads used for the reassembly were trimmed and quality filtered as already mentioned for the phylogenetic analyses. We discarded sequences with a coverage lower than three reads.

The SNPs obtained through the pipeline were subsequently filtered, eliminating all positions with >35 % of missing data. In addition, if two or more SNPs were ≤25 positions apart, only one was selected, prioritizing the one with the lowest percentage of missing data.

### Single nucleotide polymorphism-based analyses

The SNP dataset was analysed with the multispecies coalescent method SVDQuartets (Chifman and Kubatko, 2014) implemented in PAUP* v.4.0a146 (Swofford, 2002). This method implements a site-independent molecular evolutionary model, allowing each SNP to have its own different underlying gene trees. We used the option of evaluating 20 000 quartets or all possible quartets if <20 000. Clade support was evaluated by running 100 bootstrap replicates, using the multispecies coalescent model and without assigning lineages to the individuals.

The multivariate method of discriminant analysis of principal components (DAPC) (Jombart *et al.*, 2010) was performed with the *adegenet* package v.2.1.3 (Jombart and Ahmed, 2011) in R v.4.0.4, without a priori clusters. The *find.clusters* function included in the same package was used to determine the optimal value of *K*, running the *k*-means cluster algorithm with increasing values of *K* and computing a Bayesian information criterion for each value of *K*. An optimal value of *K* = 8 was obtained. We ran the DAPC using these eight clusters, 40 principal components and seven discriminant components. A minimum spanning tree using genetic distances between clusters was also calculated with the *adegenet* package, to determine the proximity between the estimated clusters.

The genetic structure within *E. balsamifera* was explored using the Bayesian population genetic software STRUCTURE v.2.3.4 (Pritchard *et al.*, 2000), with 100 000 repetitions and a burn-in period of 10 000, assuming correlated allele frequencies and using the admixture model. The analysis was repeated using different population sizes (*K*) from 1 to 15, and repeating the analysis for each *K* ten times. The best value of *K* was determined with Clumpak (Kopelman *et al.*, 2015) using the Evanno method (Evanno *et al.*, 2005). This analysis showed a clear division into three groups: an eastern group composed of populations from Lanzarote, Fuerteventura and Gran Canaria; a western group including populations from La Gomera, El Hierro and La Palma; and a third group consisting of samples from eastern Tenerife. Additionally, we performed two subsampling analyses within the eastern and western groups, using the same parameters as above. The analysis for the western group included five populations from La Palma, El Hierro and La Gomera, and that of the eastern group included seven populations from Fuerteventura, Lanzarote and Gran Canaria. Populations from Tenerife were not included in these analyses because their inclusion each time resulted in a *K* = 2 split between the genotype of Tenerife and a single genotype for the other islands, preventing detection of the fine-scale population structure (results not shown).

Genetic diversity statistics, including allelic richness (*A*_r_), observed heterozygosity (*H*ₒ), expected heterozygosity (*H*ₑ) and Shannon diversity, were calculated using the SNP dataset with the R package DartR (Gruber *et al.*, 2018). Values of Tajima’s *D* were estimated using the exon-aligned matrices in DnaSP v.6.0 (Rozas *et al.*, 2017).

### Convolutional neural network analysis

**Fig. 2.**
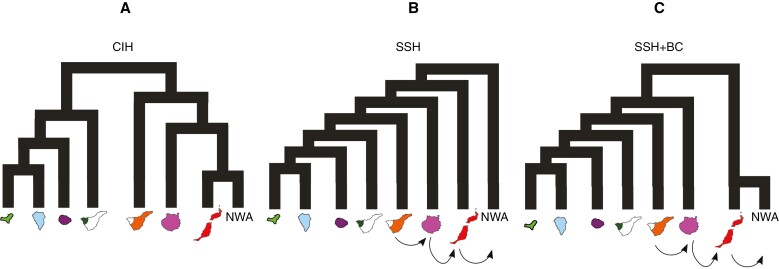
Representation of alternative colonization models of the Canary Islands tested in our study with convoluted neural networks. (A) Central island hypothesis (CIH). (B) Surfing syngameon hypothesis (SSH). (C) Surfing syngameon hypothesis including back-colonization (BC) to north-west Africa (NWA) from the islands of Lanzarote and Fuerteventura. Arrows between islands in B and C indicate migrations between connected islands after initial colonization.

For the phylogenetic models, we considered each island as a single population. The only exceptions were Lanzarote and Fuerteventura, because phylogenetic analysis based on DNA sequences and SNPs showed that individuals from these islands are intermingled and embedded in a single clade without any geographical structure (see Results). and Tenerife, which was separated into eastern and western populations. This, together with their geological history (i.e. they were united into a single island at different times), led to their inclusion in our analyses as a single population. The populations on the north-west African coast were included in the analysis and treated as a ‘continental-mainland’ population. In total, we tested the following three colonization models or scenarios, described in the Introduction and shown in Fig. 2.

The CIH model (Fig. 2A) shows Tenerife as the first island to be colonized, with an internal east/west division within the island. Subsequently, in a stepping-stone fashion, populations from western Tenerife colonized La Gomera, La Palma and El Hierro, and populations from eastern Tenerife colonized Gran Canaria, Lanzarote and Fuerteventura. Given that our results showed the north-west African populations embedded in the clade grouping Lanzarote and Fuerteventura populations, we included in this model the possibility of a back-colonization event from the eastern islands to north-west Africa.

The second scenario is the SSH model (Fig. 2B), which posits an east-to-west stepping-stone colonization of the Archipelago, starting from the continent (north-west Africa). The SSH model predicts secondary migration events in the opposite direction to the initial colonization, from the central islands to the eastern islands, with subsequent introgression between the original and recently dispersed populations (Caujapé *et al.*, 2011). According to these authors, the most likely time for these secondary migrations was during the ‘westerlies inversion’, which occurred between 31 000 and 13 000 years ago and caused the wind to blow from the Canary Archipelago towards the mainland (Rognon and Coudé-Gaussen, 1996). However, our molecular age estimates for the divergence of the eastern island populations are considerably older. Therefore, we extended the time interval for modelling migration events between 1.25 Ma and 14 000 years ago. This interval spans the beginning of the mid-Pleistocene transition to the end of the Last Glacial Maximum. During this period, glaciations, which had already started in the Early Pleistocene, intensified, and the sea level dropped. Therefore, it is estimated that the geographical distance between the Canary Islands and the mainland was significantly reduced during this period (Carracedo *et al.*, 2002), facilitating migratory events across the archipelago (Caujapé *et al.*, 2011).

**Fig. 3.**
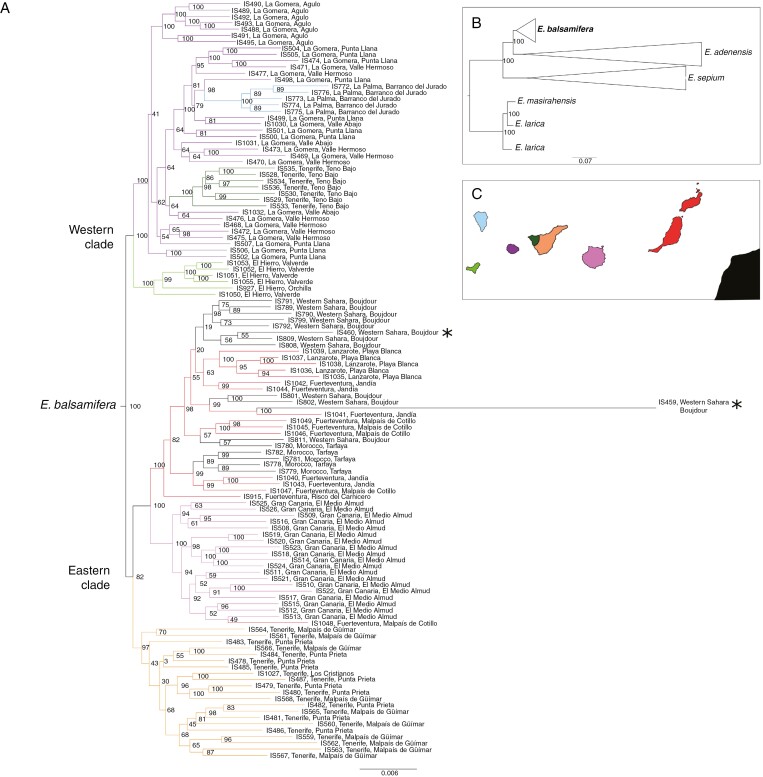
Phylogenetic relationships under maximum likelihood. (A) Phylogenetic tree showing relationships within *Euphorbia balsamifera*. The tree was obtained with IQ-TREE and is based on a concatenated supercontig matrix with 217 loci and 120 samples. Branches are coloured according to the locality (island, continent) of the individual sampled as depicted in C. Samples from north-west Africa (Western Sahara) marked with an asterisk are the herbarium samples from the study by Villaverde *et al.* (2018), which were positioned as basal in that study. (B) Schematic phylogenetic tree showing the relationship of *E. balsamifera* with its closest relatives, *E. adenensis* and *E. sepium*; the outgroup is represented by other species in *E.* section *Balsamis* (*E. larica* and *E. masirahensis*). Numbers at nodes indicate the bootstrap support values. (C) A map of the Canary Islands including the sampled area in north-west Africa.

**Fig. 4.**
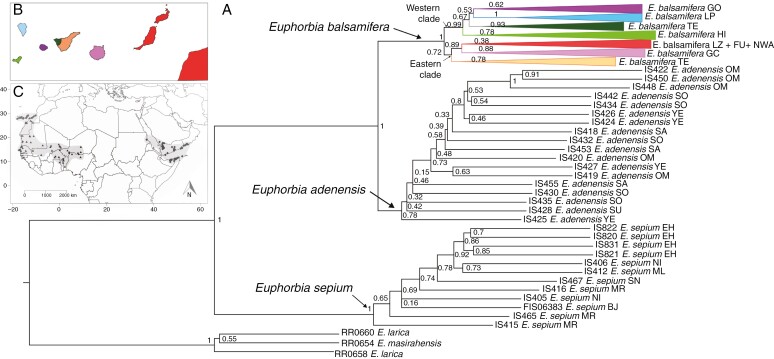
Phylogenetic relationships under the multispecies coalescent. (A) Phylogenetic tree showing relationships within *Euphorbia balsamifera* and closely related taxa. The tree was obtained with ASTRAL based on 217 gene trees from RaxML using the supercontig matrices. Collapsed clades (coloured) within *E. balsamifera* group the populations of each island, with the exception of Lanzarote, Fuerteventura and the populations from north-west Africa recovered in the same clade, and the Tenerife populations separated into two clades. Numbers at nodes indicate support values [local posterior probability (LPP)]. (B) Geographical areas/islands of the sampled populations coloured according to the collapsed clades in A. (C) Map showing the distribution of *E. balsamifera* (circles), *E. sepium* (triangles) and *E. adenensis* (diamonds). Abbreviations: BJ, Benin; EH, Western Sahara; FU, Fuerteventura; GC, Gran Canaria; GO, La Gomera; HI, El Hierro; LP, La Palma; LZ, Lanzarote; ML, Mali; MR, Mauritania; NI, Niger; NWA, north-west Africa; OM, Oman; SA, Saudi Arabia; SN, Senegal; SO, Somalia; SU, Sudan; TE, Tenerife; YE, Yemen.

**Fig. 5.**
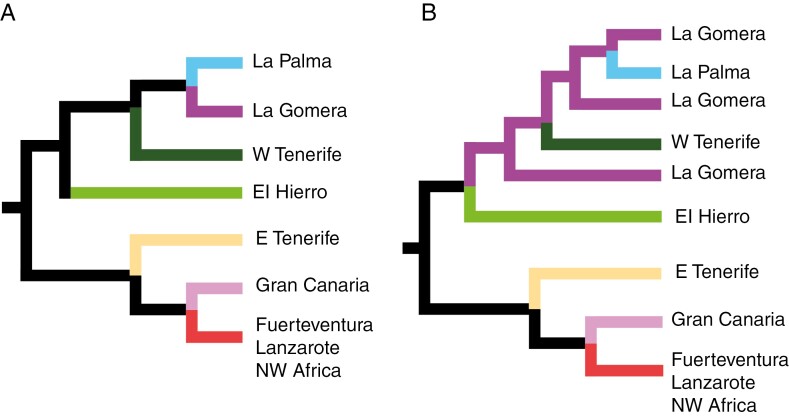
Schematic comparison of the phylogenetic analyses of *Euphorbia balsamifera* shown in Figs 3 and 4. (A) Maximum likelihood tree (ASTRAL). (B) Multispecies coalescent tree (IQ-TREE). Note that the main difference in topology is the position of the populations from La Gomera, which are monophyletic in ASTRAL but not in IQ-TREE.

The third model (SSH + BC; Fig. 2C) is a modification of the previous model, with the difference that the north-west African populations are derived from a recent colonization event from the eastern islands Lanzarote and Fuerteventura. This scenario was introduced to allow for the possibility of back-colonization in *E. balsamifera* (Villaverde *et al.*, 2018), which is also supported by our results (Figs 3–5) and has been inferred in other Canary Island endemics (e.g. Patiño *et al.*, 2015).

**Fig. 6.**
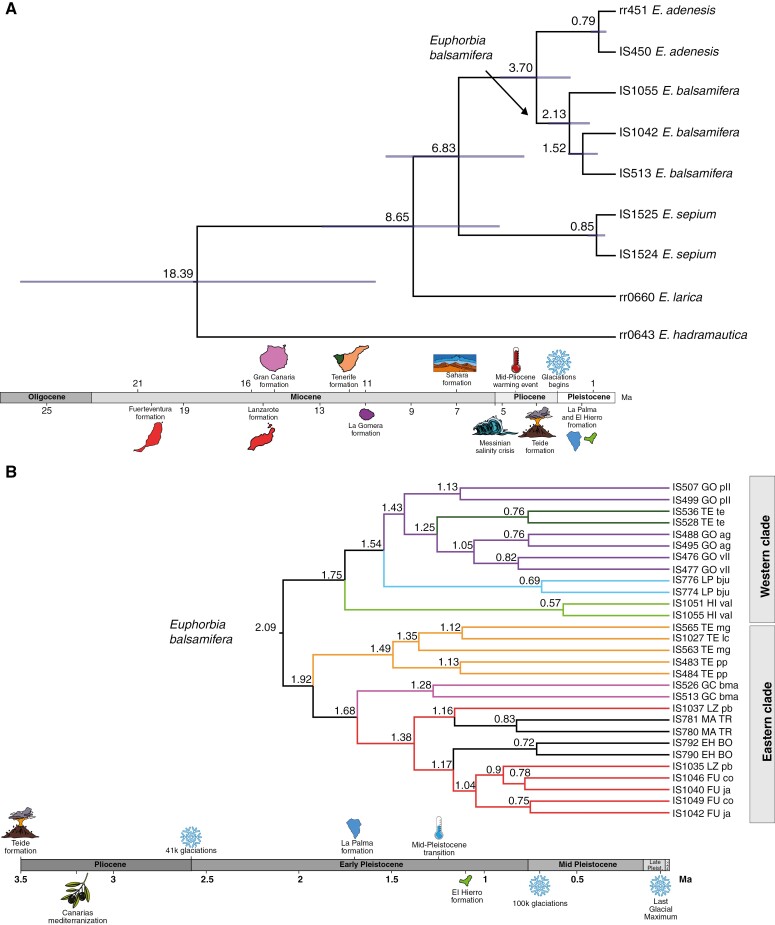
Maximum clade credibility trees with lineage divergence times estimated in BEAST using Bayesian molecular clocks. The time lines include some of the geological events that affected Africa and the Canary Islands. (A) Mean ages of divergence between *Euphorbia balsamifera*, *Euphorbia adenensis*, *Euphorbia sepium* and closely related *Euphorbia larica* and *Euphorbia hadramautica*, obtained using a Bayesian random clock and a birth–death tree prior. The node bars represent the 95 % highest posterior density (HPD) credibility intervals. The numbers above the nodes represent the dated age. The supports of each node are not shown because they all had a posterior probability of one. (B) Mean ages of divergence between populations of *E. balsamifera* obtained using a strict molecular clock and constant-size coalescent tree prior. The numbers above the nodes represent the dated age. Node support is not shown, but all values are ≥0.98. These values and the 95 % HPD credibility intervals of node ages are shown in the Supplementary Data (Fig. S10). Branches are coloured according to each island colour code, as in Fig. 3. Abbreviations: EH BO, Western Sahara, Boujdour; FU co, Fuerteventura, Malpaís de Cotillo; FU ja, Fuerteventura, Jandía; GC bma, Gran Canaria, El Medio Almud; GO ag, La Gomera, Agulo; GO pll, La Gomera, Punta Llana; GO vll, La Gomera, Valle Hermoso; HI val, El Hierro, Valverde; LP bju, La Palma, Barranco del Jurado; LZ pb, Lanzarote, Playa Blanca; MA TR, Morocco, Tarfaya; TE mg, Tenerife, Malpaís de Güímar; TE pp, Tenerife, Punta Prieta; TE te, Tenerife, Teno Bajo.

We performed 10 000 coalescent simulations for each model using the program ms (Hudson, 2002), with sample sizes according to the empirical dataset (2305 SNPs) and 1000 SNPs simulated independently (as suggested by Perez *et al.*, 2022). We used the estimated crown age (Fig. 6) as the prior for the age of the initial divergence, 2.09 Ma, with a 95 % highest posterior density (HPD) credibility interval between 0.92 and 3.38 Ma. The mutation rate (μ) used was 0.001 substitution events per million years, which is the mean rate estimated in BEAST for the set of low-copy nuclear genes used to generate the SNPs. Berg (1990) estimated the generation time of *E. balsamifera* to be between 5 and 6 years, based on direct observation of these plants. Therefore, in our analyses, we used a range sampled from a uniform prior distribution between 5 and 6 years as the generation time.

We also used a shared uniform prior for the mutation–drift balance parameter (θ) of the Lanzarote–Fuerteventura population, which ranged between one and five, based on their θ values estimated with DnaSP v.6.0 (Rozas *et al.*, 2017) on the exon-aligned DNA sequences matrices (Table 1). Values of θ for other populations were estimated as a relative ratio of the value sampled for Lanzarote–Fuerteventura, following a uniform distribution from 0.2 to 2. We then transformed the obtained θ values into the effective population size parameter (*N*_e_) using the formula θ = 4*N*_e_μ. Finally, we applied a founder effect parameter whenever a new island was colonized, with a population reduction magnitude sampled from a uniform prior distribution between 0.0 and 0.05 (following Hickerson and Meyer, 2008); this was followed by exponential growth until reaching present-day population sizes. The migration events in the SSH models (SSH and SSH + BC; Fig. 2B, C) were sampled from a uniform distribution ranging from zero to ten migrants per generation. We transformed each simulation to an image (NumPy array), including samples as columns and loci as lines. We combined the approach taken by Perez *et al.* (2022), which converts simulated genotypes into major and minor alleles, with the approach of Kirschner *et al.* (2022) to accommodate missing genotypes while training the CNN, which consists of randomly adding the observed percentages of total missing data observed in each species (4.4 %). After reshuffling the order of the arrays (simulations), we separated 25 % of random simulations to be used as the validation set, while the remaining 75 % were used as the training set. We used the network architecture from the study by Perez *et al.* (2022), modified to include suggestions from Sanchez *et al.* (2021) and Nikanjam and Khomh (2021). The suggestions included were the intercalation of convolutional layers without bias with batch normalization, maximal pooling as the down-sampling strategy, and the use of the stochastic gradient descent optimizer. Our network architecture consisted of three one-dimensional convolutional layers with a kernel size of three (the first layer had 125 neurons; the second and the remaining layers had 250 neurons each). These convolutional layers, intercalated with batch normalization, were followed by a maximal pooling step. Next, we added two fully connected layers with 125 neurons, intercalated with 50 % dropout. The output layer used a softmax function to export model probabilities. We used minibatches of size 500 and rectified linear unit activation functions (i.e. ReLUs; Nair and Hinton, 2010), and the network performance was assessed with a categorical cross-entropy loss function. Also, to avoid overfitting, we used two approaches based on the accuracy for the validation set: model checkpoint, which saves only the best model; and early stopping, which tolerates a maximum of 150 epochs without any improvement in the validation set accuracy. The trained model was calibrated using temperature scaling (Guo *et al.*, 2017), with a modified version of the scripts provided by Kull *et al.* (2019) (available at: https://github.com/markus93/NN_calibration). The trained network was used to predict the most probable model of colonization, using 100 randomly sampled datasets of 1000 SNPs from the empirical data and a new set of 10 000 independent simulations per scenario (test set) not evaluated by the network during the training.

**Table 1.** Genetic diversity statistics of *Euphorbia balsamifera* populations: allelic richness (*A*_r_), observed heterozygosity (*H*ₒ), expected heterozygosity (*H*ₑ), θ, Shannon diversity and Tajima’s *D*. The value of θ*W* estimated for the north-west African populations was obtained by pooling individuals from both populations, rather than each one separately. Abbreviations: n.d., undetermined owing to an insufficient number of sampled individuals; n.ind., number of individuals used to obtain the information in each area; n.sites, average number of sites in each of these matrices.

Population	n.ind.	*H*_o_	*H*_e_	*A*_r_	Shannon diversity	n.sites	Tajima’s *D*	θ*W*
Fuerteventura	10	0.211	0.170	1.614	1.349	1783.14	−0.059	3.544
Lanzarote	5	0.189	0.148	1.500	1.302	2039.86	n.d.	4.231
Gran Canaria	19	0.116	0.095	1.373	1.196	1712.12	−0.200	4.039
Eastern Tenerife	21	0.125	0.114	1.476	1.235	1707.52	−0.229	4.206
Western Tenerife	7	0.147	0.119	1.449	1.245	1723.71	−0.045	3.862
La Gomera	29	0.115	0.099	1.499	1.207	1703.15	−0.482	3.637
La Palma	5	0.122	0.117	1.359	1.238	1730.66	n.d.	3.066
El Hierro	5	0.115	0.079	1.225	1.159	1728.44	n.d.	3.169
Boujdour	10	0.200	0.172	1.686	1.355	1725.82	−0.074	2.739
Tarfaya	5	0.128	0.100	1.291	1.204	1767.83	n.d.	2.739

The CNN predictions were then used to perform an ABC step, as recommended by Sanchez *et al.* (2021) and used by Kirschner *et al.* (2022). Cross-validation runs were performed with ten pseudo-observed simulations per scenario to evaluate the capacity of our ABC implementation to predict the correct simulated scenario from the test set CNN predictions (10 000 simulations per scenario). The CNN predictions for the empirical data were averaged and used as input to perform a rejection step in the ABC approach, retaining the 5 % most similar simulations to approximate the posterior probability of the model, using the rejection algorithm implemented in the R package *abc* (Csilléry *et al.*, 2012). The same procedure was applied to perform parameter estimation, with the difference that only simulations from the preferred model were used and only 0.1 % of them were retained in the posterior. All scripts used to perform our CNN and ABC approaches are available at: https://github.com/manolofperez/CNN_Canarias.

## RESULTS

### Phylogenetic analyses

The phylogenetic relationships at the species level inferred using IQ-TREE and ASTRAL based on the exon and supercontig datasets were consistent in their topology and showed similar values of clade support (Local Posterior Probability (LPP) = 100, Bootstrap (BS) = 1; Figs 3 and 4; Supplementary Data Figs S6 and S7). *Euphorbia sepium* was recovered at the most basal node, sister to the clade formed by *E. adenensis* and *E. balsamifera*, which in turn were sister to each other (Figs 3 and 4; Supplementary Data Figs S6 and S7).

The sampled populations of *E. balsamifera* did not show any geographical structure within islands (i.e. individuals were not grouped per population), with the exception of Tenerife. On this island, the westernmost population, from Teno, was not grouped with the rest of the populations of the island, forming a separate clade, closer to the clades of the islands of El Hierro, La Gomera and La Palma (Figs 3 and 4). Also, in the two analyses all the populations were divided into two groups (LPP = 100, BS = 1; Figs 3 and 4): one composed of populations from the easternmost islands [eastern clade (EC); LPP = 82, BS = 0.72; Figs 3 and 4] and the other formed by populations from the westernmost islands [western clade (WC); LPP = 100, BS = 0.99; Figs 3 and 4].

Clade EC included individuals from the islands of Lanzarote, Fuerteventura, Gran Canaria and the eastern part of Tenerife (eastern Tenerife), in addition to all the populations from north-west Africa (NWA). Samples from Lanzarote, Fuerteventura and NWA were grouped together, forming a single clade in both analyses (Figs 3 and 4). The relative position of populations within the EC was the same in IQ-TREE and ASTRAL analyses (Fig. 3): the eastern Tenerife clade was sister to the rest of the EC individuals, and the clade formed by samples from Lanzarote, Fuerteventura and NWA appeared as sister to the populations from Gran Canaria.

Within WC, the samples from El Hierro were recovered as monophyletic in the two analyses (Figs 3 and 4; Supplementary Data Figs S8 and S9), whereas the positions of samples from the other islands varied among phylogenetic trees. In IQ-TREE, the El Hierro clade was sister to a clade including samples from La Gomera, La Palma and western Tenerife (LPP = 100; Fig. 3). La Gomera samples did not form a monophyletic group, but appeared to be split into several clades, with samples from Tenerife (LPP = 100) and La Palma (LPP = 100) embedded within. La Palma samples grouped into a single clade, nested with several samples from La Gomera (LPP = 98; Fig. 3); likewise, samples from western Tenerife formed a clade sister to other samples from La Gomera (LPP = 64; Fig. 3). In contrast, in the ASTRAL analysis (Fig. 4), the WC samples formed three subclades, each corresponding to one island (La Gomera, La Palma and western Tenerife), with western Tenerife (BS = 0.93) sister to the clade (BS = 0.53) containing the subclades of La Gomera and La Palma (BS = 0.67; Fig. 4). The analysis using the supercontig matrices and IQ-TREE (Supplementary Data Figs S8 and S9) placed the samples from La Palma and western Tenerife in a clade sister to the clade formed by La Gomera populations (LPP = 100; BS = 0.67), whereas in the ASTRAL analysis each island formed a single clade, with La Palma sister to La Gomera (BS = 0.53; Supplementary Data Fig. S9).

### Divergence time estimation

The tree topology recovered from the BEAST analysis at the species level (Fig. 6A) was congruent with the ASTRAL and IQ-TREE topologies based on the supercontig matrices (Figs 3 and 4). The divergence of *E. sepium* from the *E. balsamifera*–*E. adenensis* clade was dated in the Late Miocene, Messinian age (6.83 Ma, 95 % HPD 3.12–10.46 Ma), and the divergence between *E. balsamifera* and *E. adenensis* was dated around the Mid Pliocene (3.70 Ma, HPD 1.80–5.65 Ma). The divergence of the clade of these three species from *E. larica* was estimated at 8.65 Ma, in the Late Miocene, Tortonian age (95 % HPD 3.93–13.46 Ma). The second dating analysis, using the dataset representing all *E. balsamifera* populations (Fig. 6B), recovered a topology congruent with those obtained in the phylogenetic analyses (Figs 3 and 4), with a division between clades EC and WC.

As in the ASTRAL analysis (Fig. 4), samples from each island were grouped into subclades. The exceptions were the populations from Lanzarote, Fuerteventura and north-west Africa, which were placed together into a single clade, without an island-constrained geographical structure. Likewise, the individuals from western Tenerife were embedded within the clade formed by individuals from La Gomera (Fig. 6), as in the IQ-TREE analysis (Fig. 3).

### Single nucleotide polymorphism calling and population-level phylogenetic analyses

**Fig. 7.**
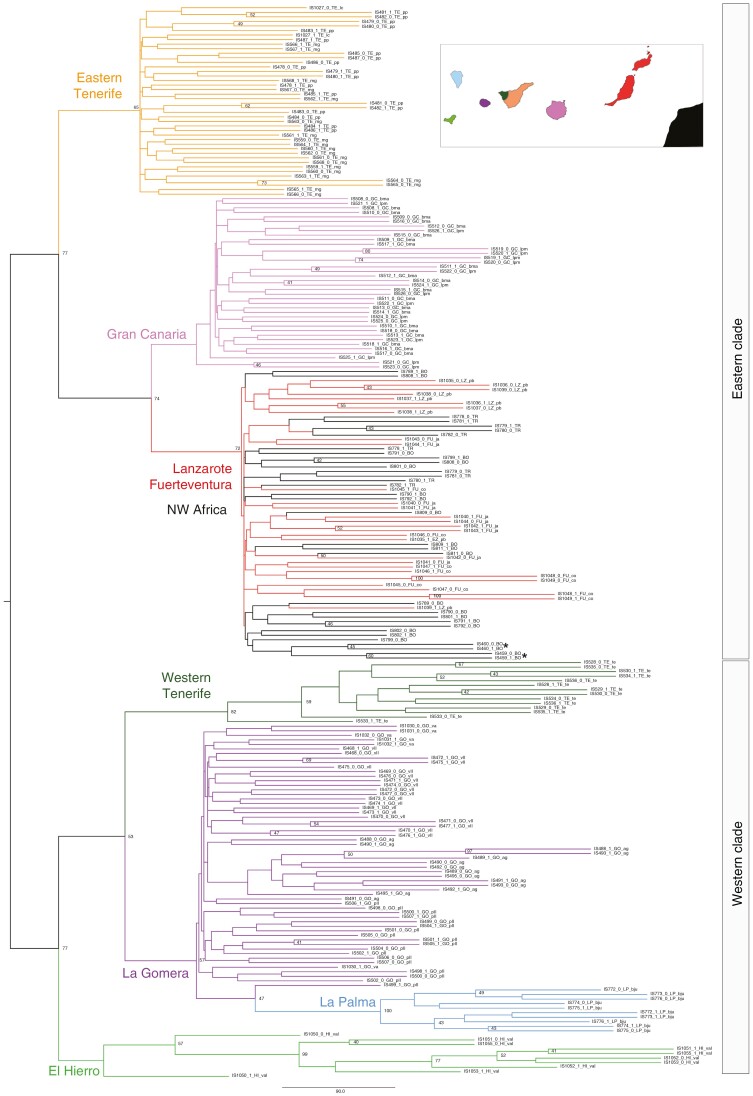
SVDQuartets phylogenetic tree based on a 2305 SNP dataset, depicting relationships among populations of *Euphorbia balsamifera*. Colours of branches correspond to those shown in the inset map of the Canary Islands. Only nodes with support values >40 % are indicated. Abbreviations: BO, Western Sahara, Boujdour; FU co, Fuerteventura, Malpaís de Cotillo; FU ja, Fuerteventura, Jandía; GC bma, Gran Canaria, El Medio Almud; GO ag, La Gomera, Agulo; GO pll, La Gomera, Punta Llana; GO vll, La Gomera, Valle Hermoso; HI val, El Hierro, Valverde; LP bju, La Palma, Barranco del Jurado; LZ pb, Lanzarote, Playa Blanca; TE lc, Tenerife, Los Cristianos; TE mg, Tenerife, Malpaís de Güímar; TE pp, Tenerife, Punta Prieta; TE te, Tenerife, Teno Bajo; TR, Morocco, Tarfaya. Samples from north-west Africa (BO) marked with an asterisk are the problematic ones from the study by Villaverde *et al.* (2018); see main text.

A total of 2305 SNPs were recovered, allowing a maximum of 35 % missing data for each SNP. The phylogenetic tree obtained with the multispecies coalescent method SVDQuartets applied to the SNP dataset (Fig. 7) was similar in topology to those obtained using the supercontig DNA sequences (Figs 3 and 4); it showed a split between EC and WC populations (Fig. 7). Phylogenetic analysis of SNP data did not differentiate between samples from Lanzarote, Fuerteventura and north-west Africa. Likewise, the population from western Tenerife was recovered as sister to the clade containing populations from La Gomera and La Palma (Fig. 7).

### Discriminant analysis of principal components and STRUCTURE analyses

**Fig. 8.**
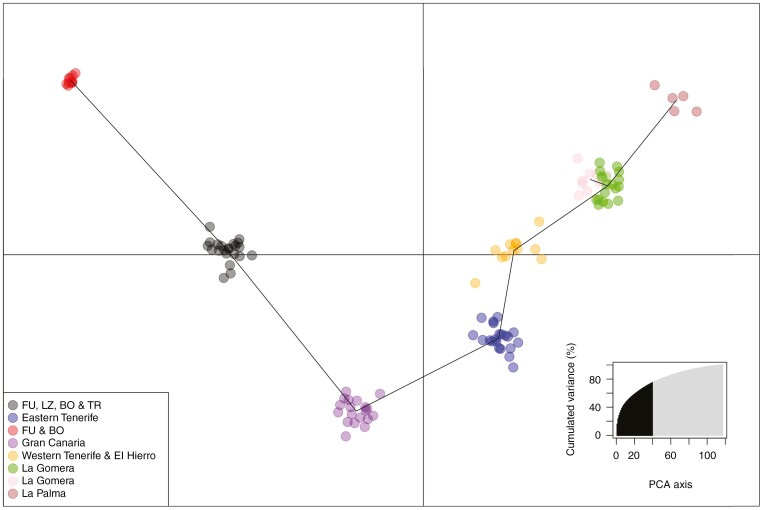
Discriminant analysis of principal components (DAPC) scatter plot showing the clustering of populations within *Euphorbia balsamifera* along the two first principal components based on the 2305 SNP dataset. Clusters were not defined a priori but were inferred using the *find.clusters* function in the *adegenet* package. The *K* value with the lowest Bayesian information criterion was *K* = 8. Lines linking the most similar populations were calculated with a minimum spanning tree, using genetic distance. The inset at the bottom left shows how the variance of the data increases as the number of principal component analysis (PCA) eigenvalues increases. In our case, 50 were selected, which represents 78 % of the data variance. Abbreviations: BO, Boujdour (Western Sahara); FU, Fuerteventura; LZ, Lanzarote; TR, Tarfaya (Morocco).

The lowest Bayesian information criterion in the DAPC corresponded to *K* = 8. The clusters found using this criterion showed geographical concordance (Fig. 8). Three of the clusters were formed by samples from a single island (La Palma, Gran Canaria and eastern Tenerife). The samples from La Gomera were divided into two clusters (northern and southern populations). The samples from Fuerteventura, Lanzarote and the mainland formed two clusters. Finally, the samples from El Hierro and western Tenerife were classified as a single group. The analysis placed the western clusters closer together and restricted to a single quadrant of the graph, while the eastern ones appeared more dispersed. The minimum spanning tree showed that eastern and western islands were interrelated, with the Tenerife samples occupying a central position.

**Fig. 9.**
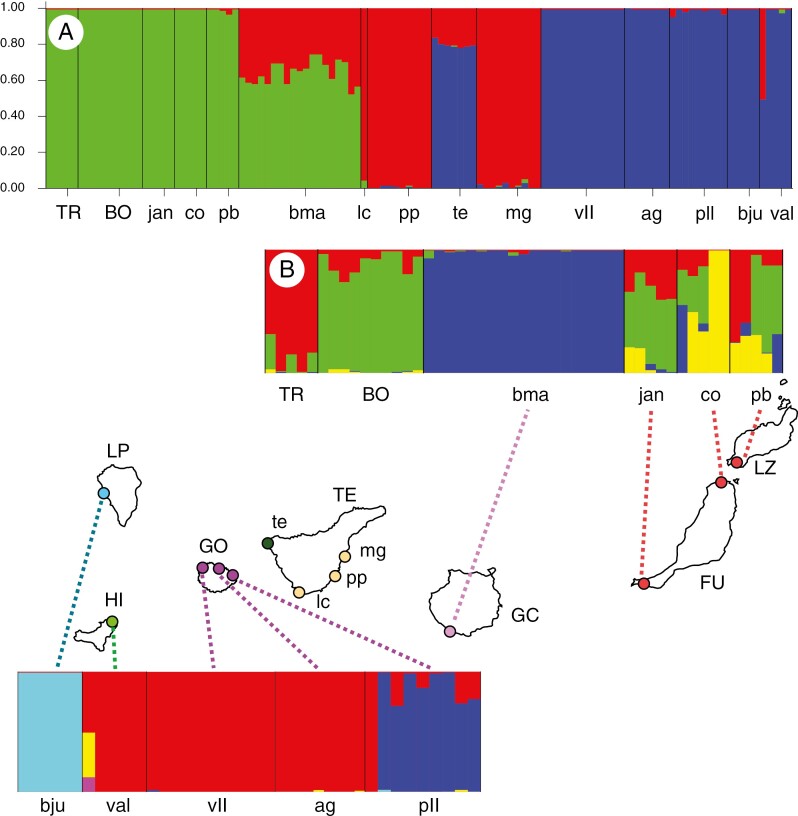
Results from the STRUCTURE analysis of *Euphorbia balsamifera* based on a 2305 SNP dataset with a maximum of 35 % missing data per locus. Each colour represents a genotype, and each vertical bar represents an individual. (A) Analyses including populations from all Canary Islands and north-west Africa (*K* = 3). (B) Analyses including populations from: (top) Lanzarote, Fuerteventura, Gran Canaria and north-west Africa (*K* = 5); and (bottom) El Hierro, La Gomera and La Palma (*K* = 4). Tenerife populations were not included in these two subsampling analyses because their inclusion each time resulted in a *K* = 2 split between Tenerife and the other islands. The inset map shows the geographical position of the populations from the Canary Islands; the north-west African populations are not shown (but see map in Fig. 1). Abbreviations: ag, La Gomera, Agulo; bju, La Palma, Barranco del Jurado; bma, Gran Canaria, El Medio Almud; BO, Boujdour (Western Sahara); co, Fuerteventura, Malpaís de Cotillo; ja, Fuerteventura, Jandía; lc, eastern Tenerife, Los Cristianos; mg, eastern Tenerife, Malpaís de Güímar; pb, Lanzarote, Playa Blanca; pll, La Gomera, Punta Llana; pp, eastern Tenerife, Punta Prieta; te, western Tenerife, Teno Bajo; TR, Tarfaya (Morocco); val, El Hierro, Valverde; vll, La Gomera, Valle Hermoso.

The Evanno method estimated *K* = 3 as the optimal *K* value for the STRUCTURE analysis including all populations. Populations within the EC and WC exhibited little admixture, with the exception of Gran Canaria (Fig. 9A), i.e. each clade had its own genotype. Eastern Tenerife also exhibited its own genotype, which showed introgression into the populations of Gran Canaria and western Tenerife. The STRUCTURE analyses performed on the WC and EC (Fig. 9B), separately, and excluding populations from Tenerife, exhibited a *K* = 4 and *K* = 6 structure, respectively. High levels of admixture were found among the eastern populations, especially within Fuerteventura (FU) and Boujdour (BO; mainland). Within the WC populations (Fig. 9B), La Palma and El Hierro exhibited their own genotypes, with negligible levels of admixture, whereas La Gomera showed some admixture between populations; a clear genetic split was found between the GO pll population and the other populations in the island. One of the individuals (IS1050) from El Hierro showed two genotypes that were not found in any other WC individual (Fig. 9B). This sample did not have more missing data than the rest of the El Hierro samples, nor did it have lower cover data (Supplementary Data Tables S2–S4). In fact, this individual was always recovered at the basal node of the El Hierro clade in the phylogenetic analyses, which, in this context, could be an indication of sequencing artefacts.

### Genetic diversity statistics

Population genetic diversity statistics grouped by island/locality showed higher *A*_r_, *H*ₒ and Shannon diversity indices in the eastern islands (Fuerteventura and Lanzarote) and in the mainland locality of Boujdour (Table 1). These diversity indices decreased towards the west, being the lowest in the western islands. The value of Tajima’s *D* was estimated as negative in all cases, suggesting an abundance of rare alleles and therefore indicative of expanding populations or allele selection.

### Convolutional neural network analysis

The percentage of correct assignments using the simulated data was >0.94 for all three scenarios (Table 2A), indicating a high level of accuracy for our CNN architecture. Once trained, we tested the efficiency of the CNN to predict the best-fitting model with our empirical genetic data (Table 2B). The model selected was SSH + BC (*P* = 0.75). As stated in the Introduction, the SSH + BC model is a modification of the SSH (i.e. east-to-west stepping-stone colonization of the archipelago from north-west Africa, allowing for secondary migration events in the opposite direction), with the north-west African populations originating from a recent colonization event from the eastern islands of Lanzarote and Fuerteventura.

**Table 2.** (A) Convolutional neural network cross-validation using the three examined colonization models. (B) Support for the selected model (SSH + BC). Abbreviations: CIH, central island hub; SSH, surfing syngameon hypothesis; SSH + BC, surfing syngameon hypothesis with back-colonization from Lanzarote and Fuerteventura to north-west Africa.

(A)	Simulated model	Predicted model		
		SSH	SSH + BC	CIH
	SSH	0.944	0.053	0.003
	SSH + BC	0.054	0.946	0.000
	CIH	0.001	0.000	0.999
(B)	Scenario	Probability		
	SSH	0.219		
	SSH + BC	0.752		
	CIH	0.0293		

## DISCUSSION

### Back-colonization of Africa rather than relicts of mainland populations

Mairal *et al.* (2015*a*) proposed that the Macaronesian element of the Rand Flora pattern was the result of recent dispersal events during the Pleistocene from populations in north-west Africa that are now extinct owing to continental aridification. This hypothesis was supported by the much younger age of Canarian Rand Flora species in comparison to their eastern African counterparts (Mairal *et al.*, 2015*a*; Villaverde *et al.*, 2018; Culshaw *et al.*, 2021; Rincón-Barrado *et al.*, 2021). Villaverde *et al.* (2018) found support for the relictual character of north-west African populations of *E. balsamifera* based on multispecies coalescent phylogenetic analysis (ASTRAL and SVDQuartets) and Bayesian phylogenetic dating approaches (BEAST): samples from south-west Morocco and Western Sahara were placed with high support as the sister clade or in an early-diverging grade relative to the island populations. This result was in conflict with the position recovered in the ML concatenated analysis (IQ-TREE), which showed these samples embedded within an insular Tenerife clade. By increasing taxon sampling in our study (adding field-collected samples from the continent and from the eastern islands), we recovered a position that was partly congruent with the IQ-TREE analysis of Villaverde *et al.* (2018): the continental samples from north-west Africa were placed within a clade that included individuals from Lanzarote and Fuerteventura. Moreover, this western–eastern topology was supported by all our analyses [IQ-TREE (Fig. 3), ASTRAL (Fig. 4), BEAST (Fig. 6) and SVDQuartets (Fig. 7)], with high support. Therefore, our results reject the hypothesis that the north-west African populations of *E. balsamifera* are climatic relicts (Pokorny *et al.*, 2015). Instead, these populations originated from back-migration events to the continent, after the initial colonization of the archipelago, probably from the nearest islands (Lanzarote and Fuerteventura), which are located ~110 km from the north-west African coasts.

In the 19th century, it was hypothesized that part of the African flora could have originated in Macaronesia and later colonized the mainland (Ball, 1878), although the view during the 20th century was to consider the islands as biodiversity ‘cul-de-sacs’ or evolutionary ‘dead-ends’ (Wilson, 1961). The molecular systematic revolution, which enabled the reconstruction of robust phylogenetic trees from DNA sequence data, and the introduction of the molecular clock to estimate divergence times have since demonstrated that back-colonizations to the African continent have not been rare in the phylogeographical history of Macaronesian lineages; examples include the angiosperm genera *Brachypodium*, Poaceae (Catalán and Olmstead, 2000); *Cytisus*, Fabaceae (Cubas *et al.*, 2002); *Aeonium*, Crassulaceae (Mort *et al.*, 2002); or *Plantago*, Plantaginaceae (Ronsted *et al.*, 2002). Likewise, fine-scale phylogeographical history inferred from patterns of genetic variance in multicopy chloroplast and nuclear markers suggests that introgression from island populations into continental populations has been more frequent than traditionally assumed (Bellemain and Ricklefs, 2008; Agnarsson and Kuntner, 2012; Patiño *et al.*, 2015). Even long-standing hypotheses, such as the loss of the dispersal capacity in island species, cannot be applied to all cases (García‐Verdugo *et al.*, 2017; García-Verdugo *et al.*, 2019). This evidence has changed the view that volcanic oceanic archipelagos, such as the Canary Islands, act as sink areas of allele immigration; instead, they represent a source of emigrant alleles, in which island propagules introduced new genetic variability into the African populations (Mort *et al.*, 2002; Carine *et al.*, 2004; Caujapé-Castells *et al.*, 2011; Hutsemekers *et al.*, 2011; Patiño *et al.*, 2015).

Although our data are not conclusive, the continental populations of *E. balsamifera* seemingly originated from several colonization events from the eastern Canary Islands. This is evidenced in the non-monophyly of the north-west African populations in the BEAST and SVDQuartet analyses and by the grouping of individuals in the DAPC: individuals from Tarfaya (Morocco) appear grouped with those of Lanzarote, whereas some individuals from Boujdour (Western Sahara) are grouped with samples from Fuerteventura (Figs 6–8). The ages of these back-colonization events fall around the end of the mid-Pleistocene transition (Loulergue *et al.*, 2008), although the large, overlapping confidence intervals in our molecular estimates (Fig. 6) preclude detailed inferences. An increase in aridity, with longer and more intense glacial cycles (the 100 000 year glaciations; Schefuß *et al.*, 2003), started around this period, which also affected northern Africa (Aït Hssaine and Bridgland, 2009). Coastal areas in north-west Africa and the Canary Islands enjoyed a more stable climate (Jolly *et al.*, 1998) and might have served as a climatic refuge for many species during the ice ages (Husemann *et al.*, 2014). In addition, the increase in global ice reduced the geographical distance from Lanzarote and Fuerteventura to Africa (as short as 65 km, in comparison to 110 km at present; Fernández-Palacios *et al.*, 2011) and could have favoured the exchange of plant propagules between these islands and the continent. Another hypothesis is that of niche pre-emption (Caujapé-Castells *et al.*, 2011): populations in Lanzarote and Fuerteventura, the two driest islands in the archipelago, and therefore pre-adapted to arid conditions, would have been able to (back-)colonize north-west Africa only when a new arid environment was established in the continent during the mid-Pleistocene.

### Macro- and micro-evolution in ‘sweet tabaiba’

Using the ability of the Hyb-Seq technique to bridge micro- and macro-evolutionary scales (Villaverde *et al.*, 2018; Johnson *et al.*, 2019; Helmstetter *et al.*, 2020; Larridon *et al.*, 2020), we were able to infer phylogenetic relationships among populations of *E. balsamifera* and between this species and its close relatives (*E. sepium* and *E. adenensis*), using a single source of genomic data. Phylogenetic relationships at the species level (Figs 3 and 4) were identical to those recovered by Villaverde *et al.* (2018). The estimated ages of divergence (Fig. 6A) were younger than in that study, but otherwise similar to those obtained with Sanger sequencing of multicopy plastid and nuclear loci (Bruyns *et al.*, 2011; Horn *et al.*, 2014; Pokorny *et al.*, 2015). The difference with respect to Villaverde *et al.* (2018) might be explained by their use of a third calibration point, the separation of *E. hadramautica* from *Euphorbia antso*, but also by the fact that these authors relied only on herbarium material for the non-*E. balsamifera* representatives; this material is typically associated with lower capture success (see Supplementary Data Tables S2 and S3), which could have biased age estimation for the most basal nodes in their phylogeny. The split between the disjunct species *E. balsamifera* and *E. adenensis* on opposite sides of the Sahara (3.7 Ma; Fig. 6) is similar to that in the study by Pokorny *et al.* (2015) (3.8 Ma) but younger than in the study by Villaverde *et al.* (2018) (4.9 Ma). It is also congruent with a period of global warming, the Mid Pliocene Climatic Optimum (4–3.5 Ma), showing the highest temperatures before industrial times (Fedorov *et al.*, 2013). The increase in aridity probably eliminated the last viable corridors between the Eastern and Western Sahara for the ancestors of *E. balsamifera* and *E. adenensis*. The fact that this split is relatively recent compared with the one found in other Rand Flora taxa (Late Miocene; Mairal *et al.*, 2015*a*; Pokorny *et al.*, 2015; Rincón-Barrado *et al.*, 2021) might be attributable to the higher tolerance to drier conditions exhibited by *E. balsamifera* and its relatives (Mairal *et al.*, 2017).

The phylogenetic trees generated from the Hyb-Seq DNA sequences show a clear east/west division among the Canarian populations of *E. balsamifera* (Figs 3 and 4). This division occurs also within the island of Tenerife, with the population on the Teno massif (west) embedded within the western clade and the remaining populations (east and central Tenerife) grouped with those of the eastern islands. This east/west phylogeographical split, with Tenerife as a ‘hinge’, has been described in other Canarian endemic species [*Canarina canariense* (Campanulaceae) (Mairal *et al.*, 2015*b*); *Phoenix canariense* (Arecaceae) (Saro *et al.*, 2015); *Kleinia neriifolia* (Asteraceae) (García-Verdugo *et al.*, 2019)] and in several monophyletic plant and animal lineages (Sanmartín *et al.*, 2008). This phylogeographical pattern does not agree well with the stepping-stone dispersal scenario (Wagner *et al.*, 1995), according to which plant propagules colonized the islands from older to younger or, as in this case, when the islands are already formed when colonization begins, from those closest to the mainland (east) to those furthest away (west). Instead, it fits better the CIH model, which predicts dispersal from the island of Tenerife towards the east and west of the archipelago (Sanmartín *et al.*, 2008). This fit, however, is not perfect. For example, one would expect the population from western Tenerife to be sister to remaining populations in the western islands. Instead, this population is embedded within the populations from La Gomera in the IQ-TREE analysis (Fig. 3). Also, in both the IQ-TREE and ASTRAL analyses, the populations from El Hierro are recovered as sister to all other populations in the western clade: west Tenerife, La Gomera and La Palma (Figs 3 and 4). This is surprising because El Hierro is the youngest island in the archipelago and is located further away from Tenerife than La Gomera or La Palma. Also, the estimated divergence time for El Hierro populations (1.75 Ma; Fig. 6) is older than the geological age of the island (1.1 Ma; Carracedo *et al.*, 1998). One explanation for this anomaly is that this age estimate corresponds to a ‘ghost’ population, which diverged within one of the older islands and subsequently colonized El Hierro; this population was either not sampled or went extinct in the source island. The grouping of populations from La Palma and West Tenerife with La Gomera populations (Fig. 3) suggests the existence of recent exchange events among these islands after the initial colonization and divergence.

Unlike other studies (e.g. Helmstetter *et al.*, 2020), which used only silica-preserved samples, part of our SNP dataset comes from sequences generated using dried herbarium samples, which confirms the efficiency of the Hyb-Seq technique to work with degraded DNA. Phylogenetic trees and clustering analyses based on this SNP dataset (Figs 6–8) support the same east–west geographical pattern obtained from the DNA sequences (Figs 3–5). Although evidence of admixture can be observed in each island (Fig. 9), the signal is stronger in the eastern clade, with introgression events between the populations of Fuerteventura and Lanzarote and those of north-west Africa (Fig. 9B). The highest heterozygosity indices are also found in the populations in Lanzarote and Fuerteventura, followed by the continental Boujdour samples (Table 1). This finding is in contradiction to the CIH colonization model, which predicts greater genetic variability on Tenerife than on the eastern islands. The purported incongruities between patterns of genetic variance extracted from the DNA sequences and the SNP dataset, and between gene trees and the population genomic analyses, might be resolved by adopting a model-based approach to phylogeographical inference. Deep learning algorithms, especially CNNs (LeCun *et al.*, 1998), have recently started to be used in phylogeography (Chan *et al.*, 2018). They have proved to be more effective than other, more traditional analyses, such as supervised machine learning random forests (Razzak *et al.*, 2018), or ABC, especially in cases where introgression is present (Flagel *et al.*, 2019). We used this approach to compare statistically the alternative models of island colonization that have been proposed for the Canary Islands (Fig. 2). The model selected, SSH with subsequent recolonization of the continent (Table 2B), explains many of the phylogeographical patterns described above. The higher genetic variability in the eastern islands (Table 1) is congruent with the SSH scenario (Caujapé-Castells *et al.*, 2011), predicting an initial colonization from the mainland to the easternmost islands, Lanzarote and Fuerteventura, followed by recolonizations by populations from east Tenerife and Gran Canaria. These latter events could also explain the observed east/west geographical split (Figs 3–7), instead of the expected east-to-west nested pattern predicted by the SSC model. Likewise, the phylogenetic position of the north-west African populations, embedded within the Fuerteventura–Lanzarote clade in all our analyses (based on either DNA sequence or SNPs; Figs 3–8), agrees well with a subsequent colonization of the continent in more recent times. In sum, based our expanded genomic sampling and statistical phylogeographical approach, the surfing syngameon hypothesis with back-colonization to continental areas (Caujapé *et al.*, 2011) is the best scenario to explain the phylogeographical history of *E. balsamifera* (Fig. 2; Table 2).

### Effect of the quality of herbarium material on Hyb-Seq efficiency at different evolutionary levels

The expanded sampling, both in the number of specimens and in geographical coverage, and the fact that most of our samples were freshly collected and silica preserved (yielding high-quality DNA extractions) are probably the factors that explain the differences found between our study and that of Villaverde *et al.* (2018). The use of DNA extracted from old, dried herbarium samples can be problematic, because the genetic material is often fragmented, contaminated with base substitutions and found in very low quantities (Drábková *et al.*, 2002; Staats *et al.*, 2011; Sayyari *et al.*, 2017; Bakker, 2018). Sayyari *et al.* (2017) argued that these fragmentary DNA data have a high impact in the estimation of gene trees, by both weakening and introducing errors in the phylogenetic signal, and that this error can mislead multispecies coalescent summary methods (e.g. ASTRAL) when estimating the species tree from the individual gene trees. Recent studies have demonstrated that Hyb-Seq is a genomic technique that can be very efficient for sequencing DNA from old herbarium samples, generally at the macro-evolutionary level (e.g. Villaverde *et al.*, 2018, 2020; Forrest *et al.*, 2019; Siniscalchi *et al.*, 2019; Gardner *et al.*, 2021). However, Forrest *et al.* (2019) warned that degraded DNA sequences generated with Hyb-Seq from old herbarium samples might appear closely related and converge in the phylogenetic tree, not because of sequence similarity but because of the type and amount of damage sustained. In particular, they observed an effect of the preservation method, with the most DNA-damaging technique generating the highest percentage of sequences with SNP artefacts.

We argue here that these artefacts, caused by low-quality DNA, are not always present and depend on the evolutionary level being tackled. For example, in our study, the herbarium samples of *E. balsamifera*, *E. sepium* and *E. adenensis* exhibited lower percentages of capture success than those coming from fresh, silica-dried samples of these species (Supplementary Data Tables S1 and S3–S5). Despite this, all these herbarium samples were correctly positioned within their respective species clades, and with high clade support in both our study (Figs 3 and 4) and that of Villaverde *et al.* (2018).

Our study also indicates that the phylogenetic method used is not as important as the quality of the sampled DNA. Recent studies have pointed out differences in consistency between supertree, concatenation approaches and coalescent-based, species tree estimation methods in phylogenomic inference (Xi *et al.*, 2016; Sayyari *et al.*, 2017; Zhang *et al.*, 2017; Wascher and Kubatko, 2021). Summary-based methods, such as ASTRAL, rely on the accuracy of gene tree estimation, hence if single gene trees contain low phylogenetic signal, species tree estimation can become unreliable (Xi *et al.*, 2016; Zhang *et al.*, 2017); conversely, SVDquartets has been claimed to be more consistent with short or uninformative markers (Wascher and Kubatko, 2021). In contrast, concatenation approaches are presumably more sensitive to non-randomly distributed missing data across genes or taxa (Xi *et al.*, 2016); for example, in the case of limited capture success from herbarium samples. In addition, concatenation approaches are less able to resolve gene tree incongruence owing to incomplete lineage sorting (for a different view, see Tonini *et al.*, 2015). These differences in performance were not observable within our *E. balsamifera* dataset. We found that by adding fresh material from the continental populations, all three phylogenetic approaches (concatenation, multispecies coalescent and Bayesian phylogenetic dating based on concatenated datasets) provided the same result, i.e. the north-west African samples appear embedded within the eastern Canary Island clade (Figs 3–7). In contrast, Villaverde *et al.* (2018), using only herbarium samples from the continental populations, obtained contradictory results between the multispecies coalescent analyses (ASTRAL and SVDQuartets) and the IQ-TREE concatenated approach, which they attributed to a long-branch attraction artefact in the latter.

Our analysis shows that the presence of long branches in highly degraded DNA samples does not necessarily result in misleading phylogenetic relationships (Sayyari *et al.*, 2017). As mentioned above, every herbarium sample of *E. adenensis*, *E. sepium* and *E. balsamifera* was recovered within their respective clades alongside the silica-dried specimens, although in almost all cases, herbarium sample branches were considerably longer than those of the silica-dried samples (Fig. 3); the same result was obtained by Villaverde *et al.* (2018). At the micro-evolutionary level, within species, information from DNA sequences from herbarium material can be reliable as long as there are other silica-dried samples with high-quality DNA from the same locations, but not when herbarium samples are the sole source of information. For example, in our analyses, the two continental individuals of *E. balsamifera* sampled by Villaverde *et al.* (2018) and used here, both associated with long branches (IS460 and IS459), were placed phylogenetically close to the silica-dried samples from the same populations by ASTRAL and SVDQuartets (Fig. 7; Supplemenatary Data Fig. S9; albeit with low support in the study by Villaverde *et al.*, 2018). An exception is IS459 in IQ-TREE (Fig. 3), where it appears grouped with a sample from Fuerteventura; this might be attributed to the long branch subtending this sequence and the sensitivity of concatenation approaches to this type of artefact compared with multispecies coalescent approaches (Rannala and Yang, 2003).

Finally, it is interesting to note the long branches of many individuals in *E. sepium* and *E. adenensis* (e.g. IS415 and IS419; Fig. 3; Supplementary Data Fig. S6). These herbarium samples have a low percentage of coverage and a high percentage of missing data (Supplementary Data Table S2). However, this alone cannot explain the long branches; other herbarium samples from these two species, such as IS435, have shorter branches but similar percentages of capture success and missing data, whereas IS419, the sample with the lowest sequence quality in our analysis, is subtended by a shorter terminal branch than, for example, (continental) samples IS459 and IS460 in *E. balsamifera*. The fact that not all samples of *E. adenensis* and *E. sepium* are subtended by long terminal branches and that only some continental samples of *E. balsamifera* are associated with short branches (Supplementary Data Fig. S6) seems to discard geographical origin (continental vs. insular species) as an explanation for this pattern. Instead, it is likely that this artefact is a result of DNA damage that modified the base composition (long indels, artefactual base substitutions, etc.; Forrest *et al.*, 2019).

## Conclusions

Our results support the Rand Flora hypothesis for the disjunct distribution of the Canarian *E. balsamifera* relative to its closest species (*E. adenensis*) on the opposite side of the Sahara Desert. They also uphold the idea of volcanic archipelagos as sources of genetic diversity, rather than as sinks of migration events, with the continental populations of *E. balsamifera* representing an example of recent recolonization from the eastern islands, in accordance with the surfing syngameon hypothesis. Finally, our study supports the efficiency of the Hyb-Seq technique to provide phylogenetic resolution at the micro- and macro-evolutionary levels using freshly collected material and herbarium material, although the latter should be used with caution.

## SUPPLEMENTARY DATA

Supplementary data are available at *Annals of Botany* online and consist of the following.

Figure S1: heatmap representing the percentage of capture for each locus and for each individual of *Euphorbia balsamifera* and its closest relatives sampled in this study. Figure S2: phylogenetic relationships under maximum likelihood, showing the relationship of *Euphorbia balsamifera* with its closest relatives, *Euphorbia adenensis* and *Euphorbia sepium*. Figure S3: phylogenetic relationships under multispecies coalescent method, showing the relationship of *Euphorbia balsamifera* with its closest relatives, *Euphorbia adenensis* and *Euphorbia sepium*. Figure S4: phylogenetic relationships under maximum likelihood, showing the relationship of *Euphorbia balsamifera* with its closest relatives, *Euphorbia adenensis* and *Euphorbia sepium*. Figure S5: phylogenetic relationships under multispecies coalescent method, showing the relationship of *Euphorbia balsamifera* with its closest relatives, *Euphorbia adenensis* and *Euphorbia sepium*. Figure S6: phylogenetic relationships under maximum likelihood, showing the relationship of *Euphorbia balsamifera* with its closest relatives, *Euphorbia adenensis* and *Euphorbia sepium*. Figure S7: phylogenetic relationships under maximum likelihood, showing the relationship of *Euphorbia balsamifera* with its closest relatives, *Euphorbia adenensis* and *Euphorbia sepium*. Figure S8: phylogenetic relationships under maximum likelihood, showing the relationship of *Euphorbia balsamifera* with its closest relatives, *Euphorbia adenensis* and *Euphorbia sepium*. Figure S9: phylogenetic relationships under multispecies coalescent method, showing the relationship of *Euphorbia balsamifera* with its closest relatives, *Euphorbia adenensis* and *Euphorbia sepium*. Figure S10: maximum clade credibility trees with lineage divergence times estimated in BEAST using Bayesian molecular clocks. Table S1: voucher information and DNA numbers of the samples of *Euphorbia balsamifera* and related taxa used in this study. Table S2: information about the percentage of capture for each nuclear locus and for each individual of *Euphorbia balsamifera* and related taxa sampled in the study. Table S3: summary of the recovery of genes from all the samples of *Euphorbia balsamifera* and related taxa used in the study. Table S4: summary of concatenated sequence of 298 genes used for aligned exons analysis for each sample of *Euphorbia balsamifera* and related taxa. Table S5: summary of concatenated sequence of 217 genes used for aligned supercontig analysis for each sample of *Euphorbia balsamifera* and related taxa.

mcae001_suppl_Supplementary_Tables_S1-S5_Figures_S1-S9
